# HIV genotypic resistance among pregnant women initiating ART in Uganda: a baseline evaluation of participants in the Option B+ clinical trial

**DOI:** 10.4314/ahs.v22i4.48

**Published:** 2022-12

**Authors:** Alexander Amone, Priscilla Wavamunno, Grace Gabagaya, Gordon Rukundo, Joyce Namale-Matovu, Samuel S Malamba, Irene Lubega, Jaco Homsy, Rachel King, Clemensia Nakabiito, Monica Nolan, Mary Glenn Fowler, Philippa Musoke

**Affiliations:** 1 Makerere University-Johns Hopkins University Research Collaboration, Kampala, Uganda; 2 Uganda Virus Research Institute, Entebbe, Uganda; 3 Institute for Global Health Sciences, University of California, San Francisco, CA, USA; 4 Department of Pathology, Johns Hopkins University School of Medicine, Baltimore, MD, USA; 5 Department of Pediatrics and Child Health, Makerere University College of Health Sciences, Kampala, Uganda

## Abstract

**Background:**

Pre-treatment HIV drug resistance is a threat to elimination of mother to child HIV transmission and could lead to virological failure among HIV-positive pregnant women. We analysed genotypic HIV drug resistance (HIVDR) of baseline samples of participants enrolled in the Option B+ clinical trial in Uganda.

**Methods:**

HIV-infected pregnant women attending antenatal care were enrolled from Uganda's National Referral Hospital (Mulago) and Mityana District general hospital and surrounding health centers (HCs). Genotypic HIV testing was performed on blood samples from the first 135 enrolled women out of a subset of 136 participants (25%) who had a baseline VL>1000 copies/mL as one sample failed to amplify.

**Results:**

159/540 (29.4%) had a VL < 1000 copies/ml and 381/540 (70.6%) had a VL >1,000 copies/ml. Of the women with VL>1000 copies/ml, 32 (23.7%) had resistance mutations including 29/135 (21.5%) NNRTI mutations, 6/135 (4.4%) NRTI mutations and 3/135 (2.2%) had both NNRTI and NRTI mutations. The most common NNRTI resistance mutations were: K103KN (5), K103N (5), V179T (4) and E138A (4).

**Conclusions:**

One quarter of the HIV-infected pregnant women in this trial at baseline had NNRTI genotypic resistance mutations. Our findings support new WHO guidelines for first-line ART that were changed to dolutegravir-based regimens.

## Introduction

Pre-treatment HIV drug resistance (HIVDR) is a threat to elimination of mother to child HIV transmission (eMTCT) and could lead to virological failure among HIV-positive pregnant women. Among HIV-infected pregnant and breastfeeding women initiating antiretroviral therapy (ART), high numbers of pre-treatment HIVDR and treatment failures have been reported^1^. Pre-treatment HIVDR refers to resistance that is detected among ARV drug-naive people initiating ART for the first time. Pre-treatment HIVDR is considered a good indicator to guide prescription of effective first line ART regiment.

Globally, there has been an increase in pre-treatment HIVDR which threatens efforts to curb the spread of HIV 1–3. Increases in both acquired and transmitted HIVDR has been reported in low-income countries over the last decade 4. For example, among pregnant women in Uganda, HIVDR in 2018 was reported to range between 5%-15% for non-nucleoside reverse transcriptase inhibitors (NNRTI) and nucleoside reverse transcriptase inhibitors (NRTI), and was <5% for protease inhibitors (PI)^5^. Given these trends, in resource-rich settings such as the U.S, drug resistance testing in drug-naïve HIV positive individuals is recommended and routinely conducted at the time of diagnosis to detect potential transmitted drug resistance 6. In high-income countries, resistance testing is recommended in women starting ART when plasma HIV RNA levels are >500 copies/mL 7. Likewise, in resource-limited settings, WHO currently recommends that scaling up of ART should be accompanied by monitoring of both pre-treatment HIVDR and resistance acquired during the course of ART 8. However, on the ground, pre-treatment HIVDR testing is rarely done in low-income countries, even in individuals with high viral load, due to high cost; and in Uganda, pre-treatment HIVDR testing is not recommended 9.

In Uganda, despite the increasing number of pregnant women initiating ART, there is limited information about pre-treatment HIVDR among these women. Documentation of pre-treatment HIVDR in HIV-infected pregnant women with detectable viral load at the time of ART initiation is important to improve the efficacy of maternal ART and infant prophylaxis. The aim of this study was to assess pre-treatment HIVDR among a subset of HIV-infected pregnant women with baseline VL>1000 copies/ml, enrolled in the Option B+ study.

## Methods

### Study design and participants

This laboratory study of resistance was nested in an open-label randomized controlled trial (RCT) (registered under Clinicaltrials.gov ID NCT02515370) conducted between May 2016 and May 2020 at Mulago National Referral Hospital, Kisenyi Health Centre IV and Kawaala Health Centre III in Kampala, the capital city of Uganda, and at Mityana District Hospital as well as Sekayonyi Health Centre IV, Mwera Health Center IV, Magala Health Center III, Naama Health Centre III and Kabuule Health Centre III in rural Mityana District, situated about 70 kms (44 miles) west of Kampala.

### Enrolment

We enrolled pregnant Ugandan women newly diagnosed with HIV according to the following inclusion criteria: age >18 years, confirmed pregnancy through clinical assessment or pregnancy test, confirmed HIV positive sero-status, willing to receive Option B+ ART for PMTCT, agreeing to come to the study clinic for scheduled appointments and to be home visited, not planning to move out of the study catchment area within the next 2 years, and willing to provide written informed consent to participate in the study. Exclusion criteria included medical, social and other circumstances that would prevent a woman from adhering to the study protocol. Eligible HIV-infected pregnant women who had initiated ART within the past 30 days and consented to the study were randomized to a standard of care (SOC) or an enhanced peer group support (intervention) arm using a computer-generated randomization list. Basic epidemiological data such as age, marital status, religion, parity and economic status were recorded at enrolment. Blood samples were taken from the participants at enrolment for real-time viral load testing (HIV-1 RNA) and plasma was stored for future drug resistance testing. We analysed HIV genotypic resistance in stored plasma obtained from a subset of the first 135 (25%) enrolled pregnant women recently diagnosed with HIV who had a baseline viral load (VL) >1000 copies/ml.

### Sample processing

Plasma was separated from whole blood by centrifuging and stored at -20oC. Stored plasma samples were separated into aliquots and VL measurements conducted in the Infectious Diseases Institute Core Lab, Kampala, Uganda, a central laboratory certified by the American College of Pathologists. Nucleic acids were extracted and tested for HIV-1 viral load using the COBAS® AmpliPrep/COBAS® TaqMan® HIV-1 Test procedure following the manufacturer's instructions (Roche Molecular Systems, Inc., Branchburg, NJ, 08876 USA) 7. Subsequently, genotypic testing was conducted at Joint Clinical Research Centre (JCRC) Lab to detect HIV drug resistance mutations in the 135 blood samples thus processed.

### RNA extraction and PCR amplification

Viral RNA was extracted from plasma samples using a QIAamp viral RNA Mini Kit (Thermo Fisher Scientific) according to the manufacturer's instructions. Reverse transcription of the HIV-1 reverse transcriptase (RT) coding region from extracted viral RNA and amplification was done with the sense primer PS2 (5′- TCCCTCAAATCACTCTTTGGCAAC-3′) and antisense primer RTA8 (5′- GCTATTAAGTCTTTTGATGGGTCATT-3′) using a Superscript III single RT-PCR system with Platinum Taq DNA polymerase kit (Thermo Fisher Scientific) as per the manufacturer's instructions. Nested PCR was done using the sense primer RTS1AD (5′- CTGTACCAGTAAAATTAAAGCCAGG-3′) and antisense RTA4 (5′- CTGTATATCATTGACAGTCCAGCT-3′) using a Superscript III Platinum Taq kit (Thermo Fisher Scientific) as per the manufacturer's instructions to generate amplicon of 800 base pairs. The amplicon was purified using ExoSAP-IT enzyme (Thermo Fisher Scientific) and quantified using a Qubit fluorometer (Thermo Fisher Scientific).

### Genetic analysis by Sanger sequencing

The cleaned PCR amplicon was then sequenced using BigDye Terminator v3.1 Cycle Sequencing Kit (Thermo Fisher Scientific). Samples were run on the ABI 3730xl genetic analyser. The obtained sequences were then analysed using RECall (REcall.bccfe.ca), an online sequence editing software. Mutation calling was performed using the Genotypic Resistance Interpretation Algorithm from the Stanford University HIV Drug Resistance Database (http://hivdb.stanford.edu) to infer the levels of susceptibility to the reverse transcriptase coding region.

### Statistical analysis

STATA software (StataCorp LP, 4905 Lakeway Drive, College Station, TX, USA) was used for analysis of sociodemographic variables.

### Ethics and consenting of participants

The study was approved by the JCRC Institutional Review Board in Uganda, the Uganda National Council for Science and Technology (UNCST), and the Johns Hopkins University (JHU) and University of California San Francisco (UCSF) Ethics Committees. Written informed consent was obtained from all study participants before being enrolled in the study.

## Results

### Demographic and clinical characteristics of the participants

A total of 540 HIV infected pregnant Ugandan women were enrolled in the Option B+ study. Their socio-demographic characteristics are presented in Table 1. The median age of participants was 25.9 years (IQR: 20.8–31 years). The majority of women, 430/540 (79.6%) were married/living together with a partner and 352/540 (65.2%) had disclosed their HIV status to their partner and or another person. At baseline, 381/540 (70.6%) women had VL>1,000 copies/ml and 159/540 women (29.4 %) had a VL<1000 copies/ml.

**Table 1:** Socio-economic characteristics of pregnant women enrolled in the Option B+ study (N=540)

Characteristic	N (Frequency)	Percent
**Study site**		
Kampala	401	74.3
Mityana	139	25.7
**Age-group (in years)**		
18–24	259	48.0
25–34	241	44.6
35+	40	7.4
Median age	25.9	IQR: 21–30
**Marital status**		
Never married	86	15.9
Married/ living together	430	79.6
Separated/divorced/widowed	24	4.5
**Religion**		
Catholic	202	37.4
Protestant	123	22.8
Muslem	112	20.7
Pentecostal/Seventh Day Adventist/ Other	103	19.1
**HIV status disclosure**		
Not disclosed	188	34.8
Partner only	149	27.6
Another person(s) only	134	24.8
Partner & Partner & another person(s)	69	12.8
Monthly income group (in USD*)		
Don't know	208	38.5
≤$137 USD (or ≤UGX 500,000)	237	43.9
>$137 USD (or >UGX 500,000)	95	17.6
**Highest level of education**		
University/College/Tertiary	25	4.6
Secondary	258	47.8
Primary	234	43.3
No formal education	23	4.3
Viral load (copies/ml)		
0–999	159	29.4
≥1000	381	70.6

** 1 USD =3,650 UG Shillings (UGX) at the time of enrolment

### Antiretroviral Drug Resistance Results

The first 136 women enrolled with a baseline VL >1000 copies/ml was selected for genotyping for ARV drug resistance. However, as one sample failed to amplify, genotyping results were available for 135 samples.

NRTI or NNRTI resistance mutations were detected in 32/135 (23.7%) of these 135 participants. Of those, 29/135 (21.5%) were NNRTI and 6/135 (4.4%) were NRTI mutations. Multiclass resistance to both NNRTI and NRTI ARVs were detected in 3/135 (2.2%) of samples. One had T225V and G1990A, one had M184MATV and K103KN and one had E44D and V179T. Three mothers had E138A, three had K103N and two had K103KN.

Pre-treatment NRTI resistance mutations that were detected included: M184V, E44D, E44ED, M184MATV, T125TAPS, T225V and V75I; and major NNRTI resistance mutations included K103KN (5), K103N (5), V179T (4) and E138A (4). Minor NNRTI mutations included E138A, E138EG, G190GE, G1990A, H221HY, K238N, K49R, V108I and V179L.

There was no difference across randomization arms in the numbers of participants with NRTI & NNRTI resistance mutations at baseline (intervention arm: 23.5%, n=16; SOC arm: 23.9%, n=16).

## Discussion

In this analysis, we found 23.7% of pre-treatment HIVDR to NNRTIs among ART-naïve HIV infected pregnant women with baseline VL > 1000 copies/ml. This finding represents the highest level of HIVDR found among ART-naïve pregnant women in Uganda to date.

According to the Uganda Population Based HIV Impact Assessment 2016–2017, HIVDR among ART-naïve pregnant women was estimated at 8.3%, though the number of samples tested was small 8. An earlier (2013) study among pregnant women initiating ART in Uganda found no resistance in a sample of 35 HIV-positive pregnant women, with the exception of minor PI resistance 9. Another study of HIVDR among an ART-naïve Ugandan population found a transmitted drug resistance rate of 7% prior to 2010 10. A large-scale study in rural Uganda documented an increasing prevalence of pre-treatment HIVDR from 1.8% to 7% during 2005–2013 among ART-naïve women but not in men 11. And Uganda data from 2018 indicated NNRTI-resistant mutant variants detected in 5–15% of samples tested.

Our findings of pre-treatment HIVDR are also higher than those reported in some other African settings but similar to findings reported in studies in western Kenya 12 and Tanzania. Wilhelmson et al. (2018) found that 10.4% of ART-naïve pregnant women with detectable viral load in Guinea Bissau had pre-treatment HIVDR to NNRTIs 2. The high level of NNRTI resistance in our study may have resulted from an increase in acquisition of HIV-resistant strains in this population. Repeated short-term Nevirapine exposure based on PMTCT regimens used in previous pregnancies could also contribute to the high NNRTI resistance.

In this study, we found that the common NNRTI mutations were K103KN, K103N (both are non-polymorphic mutations causing high level resistance to Efavirenz and Nevirapine), V179T (non-polymorphic mutation occasionally selected in patients receiving NNRTIs) and E138A. This is similar to findings from Tanzania where common mutations of K103N and Y181C were identified to conferring resistance to NVP in 35% (7/20) and 30% (6/20) of postpartum women respectively 13. These NNRTI mutations are expected since Nevirapine and Efavirenz have formed part of first-line ART in Uganda long before Option B+ was rolled out in 2012; and given that high level NNRTI resistance can occur with single mutations. Hopefully, the frequency of NNRTI resistance will decrease with the switch to INSTI-based ART, (i.e. Dolutegravir-ART) as first line therapy for HIV-infected pregnant women. The common pre-treatment NRTI resistance mutations (4% prevalence) that we found in this study include: M184V (which is selected by 3TC/FTC); as well as E44D, E44ED, M184MATV, T125TAPS, T225V and V75I.

A major strength of this study included the ability to determine baseline resistance mutations in a recent large sample of ART naïve pregnant women who were initiating antiretroviral therapy in both urban and rural populations in Uganda. There are however also caveats to this study. Study sites were located only in Central Uganda (Kampala and Mityana), and therefore our findings may not be generalizable to the whole country. The dependence on self-reported ART-naivety by the women prior to being enrolled in the study could have underestimated the number of participants who had prior ART exposure 14.

In conclusion, we found that there was a high prevalence of HIVDR among pregnant women initiating ART for Option B+ in Central Uganda. The presence of baseline resistant virus among newly diagnosed HIV-infected pregnant women may lead to increased vertical transmission and negatively impact on global efforts to eliminate new pediatric HIV infections. In addition, perinatally HIV-infected children who acquire resistant HIV strains will raise challenges for providing effective antiretroviral combinations as 1st line therapy and in the long-term.

Our findings thus provide strong support to the 2019 WHO guidelines for first-line ART that was changed from efavirenz- to dolutegravir-based regimens 15.

## Figures and Tables

**Table 2:** Resistance to Antiretrovirals (N=135)

	Percentage	Numbers	DR mutations detected
Successfully amplified	(99.3)	135	
Any	23.7	32	
NNRTI	21.5	29	K103KN (5), K103N (5), V179T (4) and E138A (4)
NRTI	4.4	6	M184V, E44D, E44ED, M184MATV, T125TAPS, T225V
NRTI and NNRTI	2.2	3	T225V and G1990A, M184MATV and K103KN, E44D and V179T

## Role of the authors

AA wrote the original draft. GR contributed to data curation. AA, PW, GG, SM, RK, JH, CN, MN and PM reviewed and edited the manuscript. SM carried out statistical analysis. JNM, IL, RK, JH, CN, ZN, MN, MGF and PM contributed to the conception and design of the study. All authors reviewed and approved the final manuscript.
